# Improving the Reliability of Optimal In-Feed Amino Acid Ratios Based on Individual Amino Acid Efficiency Data from N Balance Studies in Growing Chicken

**DOI:** 10.3390/ani3030558

**Published:** 2013-06-26

**Authors:** Christian Wecke, Frank Liebert

**Affiliations:** Division Animal Nutrition Physiology, Department of Animal Sciences, Georg-August-University Göttingen, Kellnerweg 6, 37077 Göttingen, Germany; E-Mail: cwecke@gwdg.de

**Keywords:** growing chicken, N balance studies, ideal protein concept, ideal amino acid ratio, amino acid efficiency, modeling of N utilization

## Abstract

**Simple Summary:**

Dietary amino acid concentration should closely meet the quantitative requirement of animals dependent on genotype, gender, age, aimed performance and housing conditions. Both under- and over-supply yield impaired efficacy of individual amino acid utilization and increase the nitrogen excretion. Hence, for optimal feed formulation, a validated knowledge about adequacy of dietary amino acid balance is necessary. Present studies contribute toward ensuring ideal amino acid ratios in diets for growing broiler chicken making use of a new amino acid efficiency-based procedure.

**Abstract:**

Three consecutive nitrogen balance experiments with fast-growing male broiler chickens (ROSS 308), both during starter and grower periods, were conducted to determine the ideal ratios of several indispensable amino acids relative to lysine. The control diets based on corn, wheat, fishmeal, field peas, wheat gluten and soybean oil were formulated by computer optimizing to meet the assumed ideal amino acid ratios and to fulfill both the energy and nutrient requirements of growing chicken. According to principles of the diet dilution technique, balanced control diets were diluted by wheat starch and refilled by crystalline amino acids and remaining feed ingredients, except the amino acid under study. The lysine, threonine, tryptophan, arginine, isoleucine and valine diluted diets resulted in significantly lower protein quality as compared to control diet, especially following increased dietary lysine supply (experiments II and III) and stronger amino acid dilution (experiment III). Accordingly, the limiting position of individual amino acids was confirmed, and the derived amino acid efficiency data were utilized to derive ideal amino acid ratios for the starter period: Lys (100): Thr (60): Trp (19): Arg (105): Ile (55): Val (63); and the grower period: Lys (100): Thr (62): Trp (17): Arg (105): Ile (65): Val (79).

## 1. Introduction

According to physiological needs, absorbed amino acids (AA) should be available for tissue protein synthesis in time and in a ratio that matches the AA requirement of individual tissues. Finally, recommended optimal dietary AA ratios (ideal protein concepts) summarize these tissue needs for the whole animal. However, both due to variation of individual tissue growth depending on age or performance and the ratio between AA needs for maintenance and performance, the optimal dietary AA ratio cannot be expected as constant. A maximized correspondence between dietary supply and physiological requirements is the tool to improve metabolic efficacy within the physiological possibilities and sustainability of nutrient conversion in systems for food producing animals.

However, both improved AA requirement data and procedures to evaluate the feed potential are needed. Current focus on ileal digestible AA as a tool to describe the feed potential does not take into account that on average, 40 percent of the absorbed AA could be catabolized by the enterocytes in the gut [1]. Possibilities and limitations of different procedures for AA requirement studies are discussed elsewhere [2,3,4,5,6]. A controversy exists according to the procedures, which are mostly adapted to the physiological conditions in the animal. Currently, such procedures utilize dose response studies making use of graded AA supplementation [7,8] or derive optimal ratios from AA response due to individual AA deletion from an AA complete diet [9]. According to Baker [8], diets are formulated to meet the recommendations of the National Research Council (NRC) [10], except for the amino acid under study. Estimates of the AA requirement are conducted in terms of “subjective” and “objective” estimates of the required AA concentration in the diet. The “subjective estimate” is concluded from broken-line analysis only. Deriving the first point at which the quadratic response curve intersects the plateau delivered from broken-line analysis provides the “objective estimate”. The latter is utilized for further conclusion of ideal dietary AA ratios. 

In addition, Wang and Fuller [9] have developed another approach based on a created AA complete diet near to the actual assumptions for an optimal dietary AA ratio. This complete diet contained crystalline AA supplementations for each of the AAs under study to supply 100% of the assumed requirement level. In the next step, the AA under study was individually deleted from the complete diet. Both responses were measured, due to the complete and the deleted diet, respectively. The observed slope of the response criteria between these diets was utilized for conclusion of optimal dietary ratios between individual AAs. The principle of this approach was utilized in our procedure, but the effect of individual AA deletion was directly measured by AA efficiency from modelling of observed N balance data in growing chicken [5]. The aim of present experiments was to apply observed AA efficiency data of lysine, threonine, tryptophan, arginine, isoleucine and valine for conclusions about optimal AA ratios for these AAs in diets of growing chicken.

## 2. Materials and Methods

The experiments were conducted at the Division Animal Nutrition Physiology of Goettingen University and approved by the Animal Welfare Committee of the Department for Animal Sciences. 

### 2.1. Animals and Housing

At three assigned times, day-old meat-type male chickens (ROSS 308) from a commercial hatchery were kept in a floor pen and prepared for the consecutive N balance studies by feeding a standardized starter diet up to the beginning of the two experimental periods, respectively. Starting at day 6 (starter period) and day 20 (grower period) 35 birds each (*i.e.*, 70 birds per experiment) were individually housed in metabolism cages and randomly allotted to the experimental diets (see footnote of Table 2). Housing temperature was adjusted at 32 °C (d 1) and, subsequently, continuously decreased to 22 °C at the end of the experiments (d 35). Warm red light was provided for 23 h/d. 

### 2.2. Diets and Feeding

For applying the approach of “AA complete diet”, an average of currently available ideal dietary AA ratios with lysine as the reference AA for growing chicken was initially required. Diet composition was based on conventional feed ingredients and aimed for an AA pattern near to actual assumptions about an ideal AA ratio. Statistical analysis of reported data (AA recommendations for starter and grower diets) excluded such single values, which exceeded or remained below 10 percent of the calculated average for individual AA (Table 1). Formulation of the balanced control diets (CD) for each of the three experiments aimed at meeting these assumed ideal AA ratios for both starter and grower periods. During the extended time over which these experiments were conducted, we were not able to use identical batches of feed ingredients, and so, we had to order new single feed batches. Also, each consecutively conducted experiment was designed making use of the results of the preceding experiments. The analyzed new data were applied for repeated computer optimization to fulfill the postulated ratios within ideal AA ratios (IAAR), according to Table 1. Based on the results of Experiment I, the lysine content in all diets used in Experiment II and Experiment III was elevated. In Experiment III, it was decided to reduce the isoleucine and valine contents in the diluted diets (see DD in Table 7), as compared to the concentrations used in Experiments I and II, to ensure the limiting position of these AA in the diluted diets. Table 2 provides an overview about the intended limiting position for individual AAs of Experiments I–III based on CD with different feed ingredients (Table 3) and AA concentrations as summarized in Table 6, Table 7.

Making use of the feed optimization program Fumi for Windows 3.1 (HYBRIMIN^®^ Computer+Programme GmbH & Co. KG, Hessisch Oldendorf, Germany), diets based on corn, wheat, fishmeal, field peas and wheat gluten, including small quantities of supplemented methionine, cystine and arginine, were created (Table 3). According to optimization, the final ingredient composition of all formulated CD fulfilled the postulated optimal AA ratios (values in parentheses of Table 4, compared to data in Table 1), except for histidine, leucine and phenylalanine, respectively. Consequently, these AAs remained in relative excess and had to be deleted from the current data evaluation based on dietary AA efficiency, because their limiting position was doubtful. Due to reduced demands of broiler chickens on dietary protein and AA concentrations with increasing body weight (BW), the total protein and AA contents in the CD for grower periods were diminished by diet dilution with wheat starch (Table 3). However, the protein quality between corresponding starter and grower diets was kept constant, according to an equal proportion between all feed protein sources. 

**Table 1:** Summarized recommendation of ideal amino acid (AA) ratios in diets for growing chicken.

	Ideal dietary ratios for individual AAs related to LYS *		
	n **	Average *	Standard deviation
Lysine (LYS)	26	100	0
Methionine (MET)	22	40	4
Methionine + Cysteine (MET + CYS)	24	74	2
Threonine (THR)	24	66	3
Tryptophan (TRP)	22	16	1
Arginine (ARG)	25	105	4
Histidine (HIS)	12	34	4
Isoleucine (ILE)	24	69	4
Valine (VAL)	21	80	4
Leucine (LEU)	12	110	6
Phenylalanine (PHE)	8	66	3
Phenylalanine + Tyrosine (PHE + TYR)	9	120	7

* Based on reference list number 8, 10–23; ** number of values averaged.

**Table 2:** Overview about diets with designed limiting position of individual AAs as utilized for assessment of AA efficiency within Experiments I–III. LAA, limiting AA.

Diet	LAA	Assessment of AA efficiency		
		Experiment I *	Experiment II *	Experiment III **
LYS	Lysine	x	x	x
THR	Threonine	x	x	x
TRP	Tryptophan	x	x	n.i.
ARG	Arginine	x	x	n.i.
ILE	Isoleucine	x	x	x
VAL	Valine	x	x	x

* Each of 5 birds per diet, including balanced control diet (CD) during starter and grower period, respectively; ** Each of 7 birds per diet, including balanced control diet (CD) during starter and grower period, respectively; n.i. = not investigated.

Based on the AA balanced CD, diluted diets (DD) were achieved by diet dilution with wheat starch to 80% of the supply with CD. Except in Experiment III, the dietary supply of ILE and VAL was diluted to 70% of CD to ensure the limiting position of these branched-chain amino acids (BCAAs). The remaining feed ingredients (premix, calcium carbonate, soybean oil, cellulose) and indispensable AAs were refilled up to 100% of the balanced diet (CD), except the individual AA under study. Additionally, unspecific N was refilled by supplementation of L-glutamic acid. 

### 2.3. Collection and Sampling

N balance experiments both in starter and grower periods were divided into an adaptation period (5 days) and two consecutive collection periods (5 days each), respectively. Birds were individually housed in metabolism cages with wire floors, equipped with single feeders and self-drinking systems. At the beginning of the adaptation period, the pelleted diets were supplied at the free choice level. Individual feed supply was kept constant at the beginning of the third day in the adaptation period, further adapted during the first two days of both collecting periods and kept constant again up to the end of excreta collection. Quantitative excreta collection was conducted directly from trays 2 times a day. Excreta samples were immediately frozen and stored at minus 20 °C, until further analysis.

### 2.4. Chemical Analysis

Chemical analyses were conducted according to the German standard methods [24]. Feed ingredients and experimental diets were analyzed for crude nutrients, starch and total sugars. Ether extracts of the feed were analyzed following HCl-hydrolysis. Nitrogen determination utilized the DUMAS-method (LECO^®^LP-2000, LECO Instruments GmbH, Kirchheim, Germany) and crude protein calculation by a factor of 6.25. Amino acids (except tryptophan) were analyzed by ion-exchange chromatography (LC 3000; Biotronik, Eppendorf-Netheler-Hinz GmbH, Hamburg, Germany) following acid hydrolysis without and with an oxidation step for quantitative determination of sulfur-containing amino acids. Tryptophan was quantified by reverse phase high performance liquid chromatography and fluorometric detection following alkaline hydrolysis with barium hydroxide during autoclaving (16 h, 120 °C). Additionally, apparent metabolizable energy (ME) content of balanced CD was calculated according to the World’s Poultry Science Association (WPSA) [25].

### 2.5. Model Application and Statistical Analysis

Analysis of N balance data utilized principles of an exponential N utilization model [26] and its applications for assessing the individual AA efficiency in growing animals [2,3,4,5,6,27,28,29,30,31,32]. The modeling procedure defines daily N retention in the animal depending on N intake and dietary protein quality:

NR = NR_max_T(1 − e^−b^^·^^NI^) (1)

ND = NR_max_T(1 − e^−b^^·NI^) − NMR (2)

*b* = [ln NR_max_T − ln (NR_max_T − NR)]/NI (3)

where:

**Table:** 

NR:	Daily N retention (N deposition plus NMR)	[mg/BW_kg_^0.67^]
ND:	Daily N deposition	[mg/BW_kg_^0.67^]
NMR:	Daily N maintenance requirement	[mg/BW_kg_^0.67^]
NR_max_T:	Theoretical maximum for daily N retention (Threshold value of the e-function)	[mg/BW_kg_^0.67^]
NI:	Daily N intake	[mg/BW_kg_^0.67^]
***b***:	Slope of the N retention curve (Model parameter of dietary protein quality, independent on NI)	
e:	Basic number of natural logarithm (ln).	

**Table 3:** Ingredient composition and nutrient content of the balanced control diets (g/kg as-fed).

Experiment	I	II	III	I	II	III
Age period	Starter			Grower		
Corn	430.0	400.0	380.0	395.6	370.4	352.3
Wheat	180.0	140.0	150.0	165.6	129.6	139.05
Fish meal	170.0	200.0	204.0	156.4	185.2	189.1
Field peas	130.0	150.0	150.0	119.6	138.9	139.05
Wheat gluten	40.0	30.0	40.0	36.8	27.8	37.1
Soybean oil	20.0	20.0	5.0	30.0	30.0	10.0
Premix *	10.0	10.0	10.0	10.0	10.0	10.0
Calcium carbonate	4.0	4.0	9.2	2.0	2.0	–
Cellulose	–	1.6	1.2	2.0	3.0	3.4
DL-Methionine	0.60	0.74	0.53	0.55	0.65	0.48
L-Cysteine·HCl x H_2_O	1.90	1.66	1.60	1.75	1.55	1.47
L-Arginine	1.70	–	0.17	1.56	–	0.16
Wheat starch	11.8	42.0	48.3	78.14	100.90	117.99
ME (MJ/kg)	13.1	13.5	12.8	13.4	13.8	13.1
Crude protein	229.1	239.0	239.0	210.8	221.3	221.5
Crude lipids	67.2	70.5	51.6	68.9	78.7	50.8
Crude fiber	20.0	18.6	21.2	18.5	20.3	17.6
Starch	446.4	403.8	399.5	465.7	436.7	435.9
Total sugars	32.9	24.1	26.3	33.7	35.7	32.2

* Added per kg of final diet: 2.1 g calcium, 0.8 g sodium, 5,000 IU vitamin A, 1,000 IU vitamin D_3_, 30 mg vitamin E, 2.6 mg vitamin B_1_, 4.8 mg vitamin B_2_, 3.2 mg vitamin B_6_, 20 µg vitamin B_12_, 3 mg vitamin K_3_, 50 mg nicotinic acid, 10 mg calcium pantothenate, 0.9 mg folic acid, 100 µg biotin, 1000 mg choline chloride, 50 mg Fe as iron-II-sulfate, monohydrate, 15 mg Cu as copper-II-sulfate, pentahydrate, 120 mg Mn as manganese-II-oxide, 70 mg Zn as zinc oxide, 1.4 mg I as calcium iodate, hexahydrate, 0.28 mg Se as sodium selenite, 0.55 mg Co as alkaline cobalt-II-carbonate, monohydrate and 100 mg butylhydroxytoluol.

**Table 4:** Overview about the N and AA contents of the balanced control diets (% as-fed) and the achieved dietary AA ratio (LYS = 100).

Experiment	I	II	III	I	II	III
Age period	Starter			Grower		
N	3.67	3.82	3.82	3.37	3.54	3.54
LYS	1.25 (100) *	1.35 (100)	1.35 (100)	1.15 (100)	1.25 (100)	1.25 (100)
MET	0.50 (40)	0.54 (40)	0.54 (40)	0.46 (40)	0.50 (40)	0.50 (40)
MET+CYS	0.94 (75)	1.01 (75)	1.00 (74)	0.86 (75)	0.93 (75)	0.93 (74)
THR	0.81 (65)	0.90 (66)	0.90 (67)	0.75 (65)	0.83 (67)	0.83 (67)
TRP	0.23 (18)	0.25 (19)	0.26 (19)	0.21 (18)	0.23 (19)	0.24 (19)
ARG	1.31 (105)	1.43 (106)	1.42 (105)	1.21 (105)	1.33 (107)	1.31 (105)
ILE	0.84 (67)	0.93 (69)	0.93 (69)	0.77 (67)	0.86 (69)	0.86 (69)
VAL	1.02 (81)	1.10 (82)	1.11 (82)	0.94 (81)	1.02 (82)	1.03 (82)
HIS	0.66 (52)	0.71 (53)	0.69 (51)	0.60 (52)	0.66 (53)	0.64 (51)
LEU	1.76 (141)	1.93 (143)	1.85 (137)	1.62 (140)	1.79 (143)	1.72 (137)
PHE	0.94 (75)	1.02 (76)	1.03 (76)	0.87 (75)	0.94 (76)	0.95 (76)
PHE+TYR	1.65 (132)	1.83 (136)	1.83 (136)	1.52 (132)	1.69 (136)	1.69 (136)

* Values in parentheses indicate the AA ratios.

The model parameter, *b*, is linearly dependent on the concentration (*c*) of the first limiting AA (LAA) in the feed protein [33]. The slope of the linear function (*bc^−1^*) is an expression of dietary efficiency of the limiting AA under study and is utilized as a model parameter [3,5,28]. However, the AA efficiency parameter (*bc^−1^*) summarizes absorption and post-absorptive utilization of the LAA. This application is only valid when the first limiting position of the AA under study is ensured [32]. Consequently, *bc^−1^* data from non-validated adjustment of the individual limiting position yield an overestimation of the quantitative AA requirement. According to Samadi and Liebert [4], the model parameter *bc^−1^* is directly suited for assessing optimal dietary AA ratios, because the order of observed *bc^−1^* data from individual AA is indirectly related to the physiological requirement per unit protein deposition as one important factor. In addition, AA utilization during digestion, absorption and post-absorptive utilization processing is the second important factor influencing the order of *bc^-1^* data. Accordingly, the reciprocal relationship between LYS efficiency (as reference) and the observed efficiency of the individual LAA under study is utilized to derive ideal AA ratios (IAAR):

IAAR = *bc_LYS_^−1^:bc_LAA_^−1^* (4)

Statistical analyses were run with the SPSS software package (version 19.0 for Windows, IBM SPSS Statistics, Inc., Chicago, IL, USA). Differences between variables were compared by a one-way analysis of variance (ANOVA), including Tukey and Games-Howell tests for verification of variance homogeneity and identification of statistical significance. Observations with p ≤ 0.05 were considered to be statistical significant. Individual outlier data were identified (p ≤ 0.05) according to Dixon and Massey [34]. According to the consecutive time schedule of the experiments, varying batches of ingredients and proportions, as well as some variations between nutrient and AA contents of the control diets (Table 3, Table 4), ANOVA was applied only within the individual experimental periods.

Calculations within the model utilized a uniform NMR (221 mg/BW_kg_^0.67^) and average NR_max_T data for the starter (3,884 mg/BW_kg_^0.67^) and grower period (2,972 mg/BW_kg_^0.67^) of fast growing male meat-type chickens, according to Samadi and Liebert [4]. 

## 3. Results and Discussion

Results of the conducted N balance experiments are summarized in Table 5. From these data, both dietary protein quality (*b*) and AA efficiency (*bc^−1^*) data were derived (Table 6, Table 7).

Generally, in nearly all experimental periods, lower N balance data were obtained with AA diluted diets as compared to the balanced CD, resulting from both reduced supply of individual AA and total N intakes (Table 5). A more specific evaluation of the responses due to individual AA dilution was obtained by applying the model parameter, *b*. If the limiting position of the AA under study was valid, the parameter, *b*, declined significantly. In contrast, when the optimal dietary supply exceeded the yielded response on dietary protein quality due to individual AA dilution was only marginal.

During the starter period in Experiment I, the dilution of LYS, THR and ARG led to significant impairment of the dietary protein quality (*b*) and indicated a valid limiting position of these AAs (Table 6). Other diets yielded no significant effect on *b*. On the other hand, following diets TRP, ILE and VAL, the obtained AA efficiency data (*bc^−1^*) were significantly improved. This observation could indicate a minor oversupply of these AAs in the control diet (CD), *i.e.*, the diluted diets with considerably lower contents of TRP, ILE and VAL yielded equal protein quality as compared to CD. To overcome the excessive supply of individual AAs, Experiment II utilized an elevated lysine concentration in the feed protein (Table 4). In consequence, due to the dilution diet, TRP also yielded a significantly impaired protein quality (*b*). In spite of the indicated limiting position in AA diluted diets, TRP and ARG, the efficiency of both AA were not improved (p > 0.05). It is possible that TRP and ARG act as co-limiting AAs in this CD, because no impact on efficiency (*bc^−1^*) of TRP and ARG was observed. Furthermore, this assumption is supported by significantly lower LYS efficiency in the LYS diluted diet. It cannot be excluded that changes in diet composition due to variable batches and proportions of main feed ingredients (Table 3), different dietary AA concentrations (*c*) with possibly varying AA efficiencies or the slightly lower total N contents in diluted diets (Table 6) were responsible for this unexpected observation. According to the results of Experiments I and II, diets, ILE and VAL, demonstrated that the concentrations of these BCAA were still too high to achieve the ensured limiting position. Consequently, a higher dilution of Ile and Val down to 70% of CD, as realized in Experiment III, did overcome this problem. The observed response of *b* due to diets, ILE and VAL, in Experiment III underlines that both of the AAs achieved their limiting position.

**Table 5:** Summarized data of the N balance experiments (mean ± SEM *).

Exp.	Diet	Starter period (11d–21d)			Grower period (25d–35d)		
		BW (g)	NI (mg /BW_kg_^0.67^/d)	ND ** (mg /BW_kg_^0.67^/d)	BW (g)	NI (mg /BW_kg_^0.67^/d)	ND** (mg /BW_kg_^0.67^/d)
I	CD	554 ^a^ ± 44	3,384 ^a^ ± 69	2,315 ^a^ ± 50	1,332 ± 112	2,970 ^a^ ± 67	2,010 ^a^ ± 73
	LYS	400 ^b^ ± 45	2,939 ^b^ ± 96	1,893 ^b^ ± 46	1,462 ± 52	2,633 ^b^ ± 14	1,707 ^b^ ± 29
	THR	518 ^a^ ± 55	3,160 ^a^ ± 102	2,114 ^b^ ± 51	1,370 ± 61	2,590 ^b^ ± 43	1,758 ^b^ ± 23
	TRP	528 ^a^ ± 51	3,399 ^a^ ± 138	2,297 ^a^ ± 82	1,355 ± 135	2,743 ^b^ ± 18	1,957 ^a^ ± 47
	ARG	575 ^a^ ± 37	3,137 ^b^ ± 62	2,077 ^b^ ± 65	1,147 ± 82	2,557 ^b^ ± 70	1,753 ^b^ ± 45
	ILE	597 ^a^ ± 39	3,122 ^b^ ± 64	2,238 ^a^ ± 52	1,382 ± 58	2,776 ^b^ ± 19	1,929 ^a^ ± 30
	VAL	596 ^a^ ± 41	3,121 ^b^ ± 62	2,197 ^a^ ± 63	1,416 ± 71	2,582 ^b^ ± 43	1,758 ^b^ ± 32
II	CD	624 ± 53	3,711 ^a^ ± 60	2,500 ^a^ ± 32	1,880 ± 83	3,171 ± 41	2,063 ^a^ ± 20
	LYS	625 ± 46	3,462 ^b^ ± 46	2,027 ^b^ ± 29	1,758 ± 76	3,112 ± 20	1,869 ^b^ ± 30
	THR	688 ± 49	3,513 ^b^ ± 55	2,317 ^b^ ± 43	1,759 ± 83	3,191 ± 30	1,940 ^b^ ± 25
	TRP	640 ± 50	3,367 ^b^ ± 106	2,128 ^b^ ± 60	1,847 ± 86	3,131 ± 33	1,960 ^b^ ± 33
	ARG	673 ± 49	3,470 ^b^ ± 53	2,170 ^b^ ± 43	1,777 ± 74	3,032 ± 63	1,842 ^b^ ± 35
	ILE	672 ± 52	3,456 ^b^ ± 56	2,374 ^b^ ± 46	1,804 ± 98	3,162 ± 37	1,906 ^b^ ± 22
	VAL	674 ± 50	3,462 ^b^ ± 95	2,385 ^a^ ± 85	1,840 ± 82	3,149 ± 27	1,882 ^b^ ± 36
III	CD	538 ^a^ ± 38	4,025 ^a^ ± 31	2,703 ^a^ ± 23	1,570 ^a^ ± 57	3,565 ^a^ ± 45	2,182 ^a^ ± 44
	LYS	380 ^b^ ± 32	3,506 ^b^ ± 127	2,201 ^b^ ± 95	1,475 ^a^ ± 60	3,452 ^a^ ± 86	1,912 ^b^ ± 62
	THR	468 ^a^ ± 41	3,771 ^b^ ± 83	2,503 ^b^ ± 66	1,469 ^a^ ± 52	3,449 ^a^ ± 68	2,037 ^b^ ± 45
	ILE	441 ^a^ ± 37	3,752 ^b^ ± 88	2,410 ^b^ ± 52	1,291 ^b^ ± 31	3,328 ^b^ ± 85	1,872 ^b^ ± 59
	VAL	387 ^b^ ± 34	3,459 ^b^ ± 126	2,361 ^b^ ± 77	1,309 ^b^ ± 71	2,715 ^b^ ± 167	1,562 ^b^ ± 134

* SEM = Standard error of mean; ** ND = N intake (NI) minus N excretion; ^a,b^ mean values with various superscript letters within experiment and column are significantly different from the balanced control diet (CD), respectively (p ≤ 0.05).

**Table 6:** Model parameters as derived from N balance studies with control (CD) and AA diluted (DD) diets in the starter period (mean ± SEM *).

Exp.	Diet	N-content (g/kg)	*c* (g/16g N)		*b* (b·10^6^)	*bc^−1^*	
			CD	DD		CD	DD
I	CD	36.66			314 ^a^ ± 6		
	LYS	33.93	5.47	4.73	268 ^b^ ± 3	57 ± 1	57 ± 1
	THR	34.22	3.55	3.04	292 ^b^ ± 7	88 ^B^ ± 2	96 ^A^ ± 2
	TRP	34.34	0.99	0.85	310 ^a^ ± 10	315 ^B^ ± 6	366 ^A^ ± 12
	ARG	33.56	5.72	5.00	287 ^b^ ± 8	55 ± 1	57 ± 2
	ILE	34.23	3.67	3.14	322 ^a^ ± 6	85 ^B^ ± 2	103 ^A^ ± 2
	VAL	34.17	4.46	3.83	313 ^a^ ± 8	70 ^B^ ± 1	82 ^A^ ± 2
II	CD	38.25			326 ^a^ ± 4		
	LYS	35.29	5.63	4.88	250 ^b^ ± 4	58 ^A^ ± 1	51 ^B^ ± 1
	THR	35.61	3.74	3.22	302 ^b^ ± 5	87 ^B^ ± 1	94 ^A^ ± 2
	TRP	35.75	1.05	0.90	277 ^b^ ± 6	310 ± 7	308 ± 6
	ARG	34.93	5.99	5.25	276 ^b^ ± 6	54 ± 1	53 ± 1
	ILE	35.61	3.89	3.34	321 ^a^ ± 8	84 ^B^ ± 1	96 ^A^ ± 2
	VAL	35.57	4.59	3.95	325 ^a^ ± 13	71 ^B^ ± 1	82 ^A^ ± 3
III	CD	38.24			348 ^a^ ± 6		
	LYS	38.24	5.64	4.52	284 ^b^ ± 12	62 ± 1	63 ± 3
	THR	38.24	3.77	3.02	325 ^a^ ± 11	92 ^B^± 2	108 ^A^± 4
	ILE	38.24	3.90	2.73	304 ^b^ ± 7	89 ^B^± 1	111 ^A^± 3
	VAL	38.24	4.65	3.26	321 ^b^ ± 10	75 ^B^± 1	99 ^A^± 3

* SEM = Standard error of mean; ^ab^ mean *b* data with various superscript letters within experiment and column are significantly different (p ≤ 0.05) as compared to CD; ^AB ^mean *bc^−1^* data within rows are significantly different (p ≤ 0.05).

In the grower period of Experiment I, only the diluted diets, LYS and THR, yielded a significant decline of parameter *b* (Table 7). Diluted diets, ARG and VAL, obviously tended to respond in the same direction, but according to relatively high standard errors, no significant differences were obtained. However, AA efficiency in diluted diets, TRP and ILE, was significantly enhanced. In Experiment II, due to the elevated lysine supply (Table 2) for each of the diluted diets, a significant impairment of dietary protein quality *(b)* was detected, but only the TRP efficiency in the TRP diluted diet was significantly higher (p = 0.038), as compared to diet, CD. Similar effects on model parameter *b* were observed in Experiment III, but due to the stronger dilution of diets, ILE and VAL, the response was more pronounced. In line with Experiments I and II, the efficiency of LYS and VAL was not significantly changed (p > 0.05) following dilution of these AAs.

Table 8 summarizes the results of ideal AA ratios as derived from observed individual AA efficiency dependent on experiment and growth period, respectively. Values in parenthesis indicate calculated data related to results without a significantly confirmed limiting position of corresponding AAs, except ARG and VAL in the grower period of Experiment I. In both of these cases, parameter *b* was approximately 10 percent lower compared to CD and, for the VAL diluted diet, near to an acceptable statistical significance (p = 0.056).

**Table 7:** Model parameters as derived from N balance studies with control (CD) and AA diluted (DD) diets in the grower period (mean ± SEM *).

Exp.	Diet	N-content (g/kg)	*c* (g/16g N)		*b *(b·10^6^)	*bc^−1^*	
			CD	DD		CD	DD
I	CD	33.73			473 ^a^ ± 26		
	LYS	31.20	5.47	4.73	398 ^b^ ± 9	86 ± 5	84 ± 2
	THR	31.47	3.55	3.04	424 ^b^ ± 7	133 ± 7	139 ± 2
	TRP	31.59	0.99	0.85	484 ^a^ ± 21	478 ^B^ ± 27	572 ^A^ ± 25
	ARG	30.87	5.72	5.00	431 ^a^ ± 18	83 ± 5	86 ± 4
	ILE	31.48	3.67	3.14	465 ^a^ ± 13	129 ^B^ ± 7	148 ^A^ ± 4
	VAL	31.42	4.46	3.83	426 ^a^ ± 7	106 ± 6	111 ± 2
II	CD	35.42			462 ^a^ ± 7		
	LYS	32.62	5.63	4.89	392 ^b^ ± 10	82 ± 1	80 ± 2
	THR	32.93	3.74	3.22	408 ^b^ ± 8	123 ± 2	127 ± 3
	TRP	33.10	1.05	0.90	424 ^b^ ± 10	441 ^B^ ± 7	472 ^A^ ± 12
	ARG	32.32	5.99	5.26	394 ^b^ ± 12	77 ± 1	75 ± 2
	ILE	32.93	3.89	3.35	398 ^b^ ± 6	119 ± 2	119 ± 2
	VAL	32.89	4.59	3.96	393 ^b^ ± 13	101 ± 2	99 ± 3
III	CD	35.44			474 ^a^ ± 18		
	LYS	35.44	5.64	4.52	373 ^b^ ± 15	84 ± 3	83 ± 3
	THR	35.44	3.77	3.02	419 ^b^ ± 12	126 ^B^± 5	139 ^A^± 4
	ILE	35.44	3.90	2.73	373 ^b^ ± 14	122 ^B^± 5	137 ^A^± 5
	VAL	35.44	4.65	3.26	358 ^b^ ± 30	102 ± 4	110 ± 9

* SEM = Standard error of mean; ^ab^ mean *b* data with various superscript letters within experiment and column are significantly (p ≤ 0.05) different as compared to CD; ^AB^ mean *bc^−1^* data within rows are significantly different (p ≤ 0.05).

**Table 8:** Summarized ideal AA ratios as derived from observed individual AA efficiency related to LYS (LYS = 100).

Experiment	LYS	THR	TRP	ARG	ILE	VAL
*Starter period*						
I	100	60	(16) *	100	(56) *	(70) *
II	100	61	19	110	(60) *	(70) *
III	100	(57) *	nd	nd	55	63
Mean **	**100**	**60**	**19**	**105**	**55**	**63**
*Grower period*						
I	100	62	(15) *	100	(58) *	78
II	100	65	17	110	69	83
III	100	60	nd	nd	61	77
Mean **	**100**	**62**	**17**	**105**	**65**	**79**

* Excluded from mean value, because the response of model parameter b was not significant; ** preliminary recommended IAAR; nd = not determined within the experiment.

The observed variability within the experiments and experimental periods was relatively low, as compared to literature data [10,12,17,22,59,60]. All derived IAAR were based on diets with equal native protein sources and constant protein quality over the whole experiment, respectively.

From AA requirement studies in the literature it is indicated that most of the variation in IAAR estimates is provided by differences in bird (genotype, gender, age, performance level [4,5,8,10,14,17,20,22,23,45,53,59,60]), diet (especially feed ingredients, dietary protein concentration, balance and availability of AA [4,5,8,10,46]), response parameter (e.g., BW gain, feed conversion rate, protein deposition, body composition [8,22,42,44,45,46,47,48,54,56,57,60]) and the applied mathematical model for AA requirement assessment [8,22,42,45,47]. Even if requirement data are expressed in terms of digestible AA, regardless of total *vs.* ileal (praecaecal) or true *vs.* apparent digestible, the observed considerable variation of IAAR literature data did not decline [59]. One explanation is that the concluded AA requirement data are only partly directly related to protein deposition. Additionally, assessment of the praecaecal AA digestibility meets only a part of the total utilization process. AA losses during post-absorptive utilization process, in terms of dietary AA efficiency as reported, need more consideration. Furthermore, attention must also be paid due to the nutritive interaction or antagonism between structurally similar AAs, like LYS and ARG [35], THR and GLY [36,37], TRP and large neutral AAs [38,39] and, especially, between the BCAAs, LEU, ILE and VAL [35,40], because of their impacts on the dietary AA requirement assessment and, consequently, on the derived IAAR. 

The observed ideal THR:LYS ratio was slightly below the assumption derived from literature data (Table 1), but tended to be higher in older chickens (62 *vs.* 60). These values are in a similar range as reported by Boorman and Burgess [11], Austic [13], Mack *et al*. [18] and Coon [22]. Lower THR to LYS data (56:100) were published by Baker *et al.* [7]. Likewise, Mack *et al*. [18] and Everett *et al*. [41] obtained data suggesting that the ideal ratio of THR:LYS was lower (57 or 59) than previously reported. Leclercq [42] established THR:LYS ratios between 59 and 62 to 100 based on a non-linear calculation model and BW gain, feed conversion rate and breast meat percentage as reference criteria. However, according to the “broken line” model, the calculated relative ratios were higher (63 to 65), except for feed conversion rate (only 53.5). Considerably higher values (66 to 74) were obtained in other studies [41,43,44,45,46,47,48]. The average TRP:LYS ratio of 18:100, as preliminarily recommended, was slightly higher than indicated from the mean of reference data (Table 1), but it was within the analyzed data pool, varying between 14 and 20 for the dietary needs of TRP relative to LYS (100), and agreed with the current results of Corzo [49].

Derived ratios of ARG (100 or 110) to LYS (100) were variable, but could be ranged rather in the middle of earlier estimates, indicating a very high variability between 90% and 118% related to LYS, as reviewed by Balnave and Barke [50]. Due to antagonism between ARG and LYS [51,52], the results led to the assumption that the increased ARG:LYS ratio in Experiment II could be related to the elevated dietary LYS content. Furthermore, for both ILE:LYS and VAL:LYS ratios, the current studies did not allow a final conclusion. Compared with the means of the analyzed literature data (Table 1), both calculated IAAR seem to be lower, especially in the starter period. Actual results [53] confirm this assumption. Generally, it must be concluded that the published ILE and VAL requirement estimates and derived IAAR are very variable, due to the antagonism of BCAA. If the dietary concentration of one of the BCAA was elevated, a significant increase of the requirements for the other two BCAA was observed [35,40,54]. In contrast, D´Mello [55] reported that a relatively low dietary ILE level of 0.52% permits satisfactory performance data with dietary LEU and VAL concentrations at 0.98% and 0.63%, respectively. In the majority of studies with growing chicken, especially, the LEU content in diets was very high. Consequently, both the requirements of ILE and VAL and the calculated IAAR tended to be higher [42,56,57,58].

The currently observed higher relative importance of THR, ILE and VAL in grower compared to starter diets is in line with several results derived from N balance and growth studies on broiler chickens [8,13,17,20,23,53,59].

## 4. Conclusions

The applied modelling procedure provides a new method for predicting dietary IAAR in diets for growing animals, taking into account both quantitative performance data in terms of protein deposition and the individual in-feed AA efficiency. 

Ongoing experiments with growing chicken will provide a more representative database to examine the reliability of derived IAAR. Accordingly, formulation of diets making use of the current IAAR data have to validate the IAAR conclusions based on the applied new AA efficiency procedure.

## References

[1] Wu G. (1998). Intestinal mucosal amino acid catabolism. J. Nutr..

[2] Liebert F., Benkendorff K. (2007). Modelling of threonine and methionine requirements of *Oreochromis niloticus* due to principles of the diet dilution technique. Aquac. Nutr..

[3] Samadi, Liebert F. (2007). Lysine requirement of fast growing chickens—Effects of age, sex, level of protein deposition and dietary lysine efficiency. J. Poult. Sci..

[4] Samadi, Liebert F. (2008). Modelling the optimal lysine to threonine ratio in growing chickens depending on age and efficiency of dietary amino acid utilisation. Br. Poult. Sci..

[5] Liebert F. (2008). Modelling of protein metabolism yields amino acid requirements dependent on dietary amino acid efficiency, growth response, genotype and age of growing chicken. Avian Biol. Res..

[6] Wecke C., Liebert F. (2009). Lysine requirement studies in modern genotype barrows dependent on age, protein deposition and dietary lysine efficiency. J. Anim. Physiol. a. Anim. Nutr..

[7] Baker D.H., Batal A.B., Parr T.M., Augspurger N.R., Parsons C.M. (2002). Ideal ratio (relative to lysine) of tryptophan, threonine, isoleucine, and valine for chicks during the second and third weeks posthatch. Poult. Sci..

[8] Baker D.H., D’Mello J.P.F. (2003). Ideal amino acid patterns for broiler chicks. Amino Acids in Animal Nutrition’.

[9] Wang T.C., Fuller M.F. (1989). The optimum dietary amino acid pattern for growing pigs. 1. Experiments by amino acid deletion. Br. J. Nutr..

[10] National Research Council (NRC) (1994). Nutrient Requirement of Poultry.

[11] Boorman K.N., Burgess A.D., Fisher C., Boorman K.N. (1986). Responses to amino acids. Nutrient Requirements of Poultry and Nutritional Research.

[12] Rhone-Poulenc (1993). Rhodimet Nutrition Guide.

[13] Austic R.E. Update on amino acid requirements and ratios for broiler. Proceedings of Maryland Nutrition Conference.

[14] Baker D.H., Han Y. (1994). Ideal amino acids profiles for broiler chicks during the first three weeks posthatching. Poult. Sci..

[15] CVB (1996). Aminozurenbehoefte van leghennen en leeskuikens. CVB-documentatierapport 1996.

[16] Baker D.H. (1997). Ideal amino acids profiles for swine and poultry and their application in feed formulation. Biokyowa Tech. Rev..

[17] GfE: Gesellschaft für Ernährungsphysiologie (1999). Empfehlungen zur Energie- und Nährstoffversorgung der Legehennen und Masthühner (Broiler).

[18] Mack S., Becovici D., De Groote G., Leclercq B., Lippens M., Pack M., Schutte J.B., Cauwenberghe S. (1999). Ideal amino acid profile and dietary lysine specification for broiler chickens of 20–40 days age. Br. Poult. Sci..

[19] Gruber K., Roth F.X., Kirchgessner M. (2000). Effect of partial dietary amino acid deductions on growth rate and nitrogen balance in growing chicks. Arch. Geflügelkd.

[20] Creswell D., Swick A.R. (2001). Formulating with digestible amino acids. Part two. Asian Poult. Magaz..

[21] Degussa (2003). Amino acid Recommendations for Poultry.

[22] Coon C. (2004). The Ideal Amino Acid Requirements and Profile for Broilers Layers, and Broiler Breeders.

[23] Hoehler D., Lemme A. (2005). Broiler diet formulation: Using SID. Feed Int..

[24] Naumann C., Bassler R. (1976–1997). Die chemische Untersuchung von Futtermitteln.

[25] World’s Poultry Science Association (WPSA) (1984). The prediction of apparent metabolizable energy values for poultry in compound feeds. World Poultry Sci. J..

[26] Gebhardt G., Hock A. (1966). Die Bewertung der Eiweißqualität von Nahrungs- und Futtermitteln mit Hilfe des N-Bilanzversuches. Vergleichende Ernährungslehre des Menschen und seiner Haustiere.

[27] Samadi F., Liebert F. (2006). Estimation of nitrogen maintenance requirements and potential for nitrogen deposition in fast-growing chickens depending on age and sex. Poult. Sci..

[28] Samadi F., Liebert F. (2006). Modeling of threonine requirement in fast-growing chickens, depending on age, sex, protein deposition, and dietary threonine efficiency. Poult. Sci..

[29] Samadi F., Liebert F. (2007). Threonine requirement of slow growing male chickens depends on age and dietary efficiency of threonine utilization. Poult. Sci..

[30] Liebert F., Benkendorff K. (2007). Modeling lysine requirements of *Oreochromis niloticus* due to principles of the diet dilution technique. Aquaculture.

[31] Liebert F., Wecke C. (2008). Models for further developing the evaluation of protein and amino acids as well as for predicting performance from energy and amino acids intake. Recommendations for the Supply of Energy and Nutrients to Pigs.

[32] Wecke C., Liebert F. (2010). Optimal dietary lysine to threonine ratio in pigs (30–110 kg BW) derived from observed dietary amino acid efficiency. J. Anim. Physiol. a. Anim. Nutr..

[33] Gebhardt G. (1980). Eiweiß- und Aminosäurenverwertung in Beziehung zum Stoffwechsel der limitierenden Aminosäure. Arch. Tierernähr..

[34] Dixon W.J., Massey F.J. (1957). Introduction to Statistical Analysis.

[35] D’Mello J.P.F., D’Mello J.P.F. (2003). Adverse effects of amino acids. Amino Acids in Animal Nutrition.

[36] Baker D.H., Hill T.M., Kleiss J.M. (1972). Nutritional evidence concerning formation of glycine from threonine in the chick. J. Anim. Sci..

[37] Corzo A., Kidd M.T., Dozier W.A., Kerr B.J. (2009). Dietary glycine and threonine interactive effects in broilers. J. Appl. Poult. Res..

[38] Steinhart H., Kirchgessner M. (1984). Interaction of varying supplies of tryptophan and large neutral amino acids (LNAA) on the growth of broiler chickens (in German). Z. Tierphysiol., Tierernährg. u. Futtermittelkde..

[39] Peganova S., Hirche F., Eder K. (2003). Requirement of tryptophan in relation to the supply of large neutral amino acids in laying hens. Poult. Sci..

[40] Pastor A., Liebert F. (2011). Effects of branched-chain amino acids, in particular of leucine, on protein metabolism of growing monogastric laboratory and food producing animals (in German). Übers. Tierernährg..

[41] Everett D.L., Corzo A., Dozier W.A., Tillman P.B., Kidd M.T. (2010). Lysine and threonine responses in Ross TP16 male broilers. J. Appl. Poult. Res..

[42] Leclercq B. (1998). Specific effects of lysine on broiler production: Comparison with threonine and valine. Poult. Sci..

[43] Webel D.M., Fernandez S.R., Parsons C.M., Baker D.H. (1996). Digestible threonine requirement of broiler chickens during the period three and six to eight weeks posthatching. Poult. Sci..

[44] Corzo A., Kidd M.T., Kerr B.J. (2003). Threonine need of growing female broilers. Int. J. Poult. Sci..

[45] Kidd M.T., Corzo A., Hoehler D., Kerr B.J., Barber S.J., Branton S.L. (2004). Threonine needs of broiler chickens with different growth rates. Poult. Sci..

[46] Dari R.L., Penz A.M., Kessler A.M., Jost H.C. (2005). Use of digestible amino acids and the concept of ideal protein in feed formulation for broilers. J. Appl. Poult. Res..

[47] Mehri M., Davarpanah A.A., Mirzael H.R. (2012). Estimation of ideal ratios of methionine and threonine to lysine in starting broiler chicks using response surface methodology. Poult. Sci..

[48] Mejia L., Tillman P.B., Zumwalt C.D., Corzo A. (2012). Assessment of the threonine-to-lysine ratio of male broilers from 35 to 49 days of age. J. Appl. Poult. Res..

[49] Corzo A. (2012). Determination of arginine, tryptophan, and glycine ideal-protein ratios in high-yield broiler chicks. J. Appl. Poult. Res..

[50] Balnave D., Barke J. (2002). Re-evaluation of the classic dietary arginine:lysine interaction for modern poultry diets: a review. World Poultry Sci. J..

[51] O’Dell B.L., Savage J.E. (1966). Arginine-lysine antagonism in the chick and its relationship to dietary cations. J. Nutr..

[52] Allen N.K., Baker D.H., Scott H.M., Norton H.W. (1972). Quantitative effect of excess lysine on the ability of arginine to promote chick weight gain. J. Nutr..

[53] Pastor A., Wecke C., Liebert F. Assessment of ideal dietary amino acid ratios between branched-chain amino acids for growing chickens. Proceedings of the 4th EAAP International Symposium on Energy and Protein Metabolism and Nutrition.

[54] Corzo A., Moran E.T., Hoehler D. (2004). Valine needs of male broilers from 42 to 56 days of age. Poult. Sci..

[55] D’Mello J.P.F. (1974). Plasma concentrations and dietary requirements of leucine. Isoleucine and valine: Studies with the young chick. J. Sci. Fd Agric..

[56] Corzo A., Kidd M.T., Dozier W.A., Vieira S.L. (2007). Marginality and needs of dietary valine for broilers fed certain all-vegetable diets. J. Appl. Poult. Res..

[57] Berres J., Vieira S.L., Kidd M.T., Taschetto D., Freitas D.M., Barros R., Nogueira E.T. (2010). Supplementing L-valine and L-isoleucine in low-protein corn and soybean meal all-vegetable diets for broilers. J. Appl. Poult. Res..

[58] Corrent E., Bartelt J. (2011). Valine and isoleucine: The next limiting amino acids in broiler diets. Lohmann Inf..

[59] Emmert J.L., Baker D.H. (1997). Use of the ideal protein concept for precision formulation of amino acid levels in broiler diets. J. Appl. Poult. Res..

[60] Taherkhani R., Shivazad M., Zaghari M., Shahneh A.Z. (2008). Comparison of different ideal amino acid ratios in male and female broiler chickens of 21 to 42 days of age. J. Poult. Sci..

